# Complete Genome Sequence of Japanese Vaccine Strain LA-AKO of Rinderpest Virus 

**DOI:** 10.1128/genomeA.00976-15

**Published:** 2015-09-03

**Authors:** Hitomi Takamatsu, Kazuya Terui, Takehiro Kokuho

**Affiliations:** Center for Animal Disease Control and Prevention, National Institute of Animal Health, National Agriculture and Food Research Organization, Tsukuba, Ibaraki, Japan

## Abstract

Rinderpest vaccine strain LA-AKO, which is less virulent especially to highly susceptible Asian cattle breeds, was established from a lapinized vaccine strain by further passages in rabbit and chick embryos. Here, we report the genome sequence of LA-AKO, which currently remains active for the production of an emergent vaccine in Japan.

## GENOME ANNOUNCEMENT

Rinderpest virus (RPV) is an enveloped single-stranded negative-sense RNA virus belonging to the *Morbillivirus* genus of the *Paramyxoviridae* family. RPV is highly contagious and causes severe diarrhea with high mortality in cattle, buffaloes, sheep, goats, pigs, and other species. Hence, it has been considered one of the most important pathogens for wild and livestock animals in the world. Due to the implementation of efficient vaccines combined with extensive surveillance led by international initiatives, there has been no reported field case since 2002. In 2011, FAO/OIE finally declared global eradication of rinderpest (1).

The genetic information of RPV will be useful to regenerate viruses in emergent situations in the posteradication era. However, to our knowledge, the present data deposited in public databases (Kabete O, GenBank accession no. X98291; Fusan, AB547189; RBOK, Z30697; L72, JN234008; L96, JN234010; LATC06, GU168576; LA77, JN234009; Nakamura III, AB547190) lack the information on a currently active vaccine strain, LA-AKO, which was established in Japan (2). LA-AKO originated from a rabbit-adapted vaccine strain, Nakamura III (897 passages), and attenuated further by randomly combined passages in rabbit and chick embryos (29 and 456 passages in total, respectively). This strain is less pathogenic to cattle and pigs than the parental strain, including Japanese black and Korean yellow breed cattle, which are highly susceptible to RPV infection (2, 3).

In this work, we determined the complete genome sequence of LA-AKO recovered from a final batch of RPV vaccine. Total RNAs were extracted, and overlapping cDNA fragments spanning the entire length of the genome were synthesized by reverse transcription-PCR. The PCR products were subjected to direct sequencing analysis using a conventional sequencer. The 5′ termini of the negative- and positive-sense genomes were determined by the Smarter rapid amplification of cDNA ends (RACE) kit (Clontech, USA). The complete genome of LA-AKO was 15,882 bp in length, which was identical in length to those of previously known RPVs. LA-AKO shows 98% identity with the parental Nakamura III at the nucleotide level and was classified in the same clade of Asian lineage of RPV by phylogenetic analysis (MEGA6) (4). In the sequencing analysis data, we identified mixed peaks throughout the genome, for example, at nucleotide positions 32 (T/C), 2637 (T/G), 2641 (T/C/G), and 2701 (T/A). This result indicates that the bulk vaccine presumably comprises mixed viral populations. Since it has been reported that the F protein is a major component to induce protective immunity against RPV (5), we compared the amino acid sequences between LA-AKO, Nakamura III, and the most similar strain, LA77, and identified only 13 amino acid substitutions between LA-AKO and Nakamura III and 7 between LA-AKO and LA77. The comparative analysis of genetic information in combination with the knowledge about the pathogenicity would be valuable for better understanding the attenuation mechanism of RPVs.

### Nucleotide sequence accession number.

The genome sequence of LA-AKO has been submitted to DDBJ/EMBL/GenBank under the accession number LC057619.

## References

[1] Joint FAO/OIE Committee on Global Rinderpest Eradication 2011 Final report. FAO, Rome, Italy http://www.oie.int/doc/ged/D10943.PDF.

[2] Furutani T, Kataoka T, Kurata K, Nakamura H 1957 Studies on the AKO strain of lapinized-avianized rinderpest virus I: avianization of lapinized rinderpest virus. Bull Natl Inst Anim Health 32:117–135.

[3] Nakamura J, Kuroda S 1942 Rinderpest: on the virulence of the attenuated rabbit virus for cattle. Japan J Vet Sci 4:75–102.

[4] Tamura K, Stecher G, Peterson D, Filipski A, Kumar S 2013 MEGA6: molecular evolutionary genetics analysis version 6.0. Mol Biol Evol 30:2725–2729. doi:10.1093/molbev/mst197. 24132122 PMC3840312

[5] Yilma T, Hsu D, Jones L, Owens S, Grubman M, Mebus C, Yamanaka M, Dale B 1988 Protection of cattle against rinderpest with vaccinia virus recombinants expressing the HA or F gene. Science 242:1058–1061. doi:10.1126/science.3194758. 3194758

